# Scanning Tunneling Microscopy Study of Lipoic Acid, Mannose, and cRGD@AuNPs Conjugates

**DOI:** 10.3390/nano13182596

**Published:** 2023-09-20

**Authors:** Andrés Rodríguez-Galván, Mitzi Reyes, Marisol Ávila-Cruz, Margarita Rivera, Vladimir A. Basiuk

**Affiliations:** 1 Carrera de Biología, Unidad de Biomedicina, Facultad de Estudios Superiores Iztacala, Universidad Nacional Autónoma de México, Tlalnepantla 54090, Mexico; 316225425@iztacala.unam.mx (M.R.); biomar14sol@gmail.com (M.Á.-C.); 2 Instituto de Física, Departamento de Materia Condensada, Universidad Nacional Autónoma de México, Coyoacán, Ciudad de México 04510, Mexico; mrivera@fisica.unam.mx; 3 Instituto de Ciencias Nucleares, Universidad Nacional Autónoma de México, Circuito Exterior C.U., Ciudad de México 04510, Mexico; basiuk@nucleares.unam.mx

**Keywords:** biofunctionalization, gold nanoparticles, lipoic acid, mannose, RGD cyclic peptide, scanning tunneling microscopy

## Abstract

The functionalization of AuNPs with different biological elements was achieved to investigate their possibility in biomedical applications such as drug delivery, vaccine development, sensing, and imaging. Biofunctionalized AuNPs are pursued for applications such as drug delivery, vaccine development, sensing, and imaging. In this study, AuNPs with diameters of 20 nm were functionalized with lipoic acid, mannose, or the cRGD peptide. By using UV-vis spectroscopy, Fourier transform infrared spectroscopy, dynamic light scattering, transmission electron microscopy, and scanning tunneling microscopy techniques, we showed that AuNPs can be functionalized by these biomolecules in a reliable way to obtain conjugates to explore potential biomedical applications. In particular, we demonstrate that the STM technique can be employed to analyze biofunctionalized AuNPs, and the obtained information can be valuable in the design of biomedical applications.

## 1. Introduction

Gold nanoparticles (AuNPs) are widely studied due to their interesting optical, thermal, and chemical properties that can be implemented for advanced biomedical applications [1]. In particular, they are promising platforms for molecular imaging, biomarker sensors, drug delivery, diagnostic, therapy (photon-induced), and theragnostics, among others. Several reports have shown the binding of peptides [2], proteins [3], nucleic acids [4], or carbohydrates [5] to AuNPs via ligand exchange, taking advantage of the strong thiol–gold interaction. The binding of biomolecules to AuNPs can improve their stability in physiological media and biodistribution and allows the design of conjugates with active targeting capability, which emerge due to the high specificity of biomolecules by specific biomarkers. These ligands help nanoparticles to target specific cells or tissues, improving the accumulation and biodistribution of loads (active drugs or contrast agents) compared with free loads [2,6].

Recently, the attachment of biomolecules on AuNP surfaces has been extensively studied, and several successful protocols have been reported [7]. In contrast, there is an open challenge over the orientation and assembly of the attached biomolecules. This is an important issue since the orientation, form (structure), and assembly of ligands on AuNPs can impact the biological activity and binding affinity of biomolecules; for example, the antigen-recognition Fv regions of immunoglobulin G should be correctly oriented on surfaces for effective antigen recognition [8]. Also, it is well known that the assembly of carbohydrates on cell surfaces can affect their interaction with proteins; e.g., when carbohydrates are assembled into small clusters, their binding to proteins called lectins is more efficient than when they are isolated [9], because the number of simultaneous interactions formed between clusters of carbohydrates and recognition proteins are multiple and enhance the binding affinity compared with individual carbohydrates [9]. Liese and Netz [10] reported that the interaction of synthetic systems is most efficient when the size of the clusters of recognition of biomolecules is similar compared to the size of their receptor, and, also, that an excess of ligands does not improve the selectivity, i.e., there is not a linear correlation between the interaction and the number of ligands [11].

The direct visualization of the orientation and assembly of biological ligands on the surfaces of AuNPs can help to understand and tailor their biological behavior. Several techniques have been employed for the characterization of thiolated bounded ligands on AuNPs, such as inductively coupled plasma mass spectrometry (ICP–MS), that has been used for the quantification of ligands [12], and X-ray photoelectron spectroscopy (XPS), which has been used for density packing analysis [13], and the adsorption of thiol molecules has been extensively studied using UV–visible spectroscopy (UV–vis) and in silico studies [14]. In particular, to elucidate the assembly of thiolated molecules, electron spin resonance (EPR), small-angle neutron scattering (SANS), mass spectrometry (MS), and nuclear magnetic resonance (NMR) have been employed [15]. However, for the direct visualization of biomolecules on AuNPs, scanning tunneling microscopy (STM) represents a viable alternative; for example, it has been widely employed for the analysis of biomolecules on metallic surfaces and the surfaces of nanomaterials. In particular, STM has been employed in the analysis of thiolate molecules on AuNPs and Ag nanoclusters, showing that these molecules can form patterns on AuNPs [15,16]. Also, many biomolecules such as lipids, carbohydrates, proteins, and nucleic acids have been imaged near molecular resolution on flat surfaces of Cu, Au, and highly ordered pyrolytic graphite (HOPG) [17]. In previous works, we employed STM to obtain information of individual organic molecules and proteins deposited on HOPG and the surfaces of carbon nanomaterials [18,19,20]. Also, conventional techniques employed in the analysis of biomolecules, such as circular dichroism (CD) or X-ray crystallography, present several limitations for the rutinary analysis of biofunctionalized nanoparticles, for example, the necessity of high concentrations of conjugates or the compulsory requirement of diffracting crystals [17].

By considering the importance of the orientation and assembly of biomolecules on surfaces of AuNPs, in this work, we analyzed the surfaces of functionalized AuNPs with lipoic acid (ALA@AuNPs), mannose (MAN@AuNPs), and the Arg-Gly-Asp cyclic peptide (cRGD@AuNPs). These biomolecules were selected since they might exhibit potential biomedical applications: ALA is an antioxidant biomolecule that has been used as a linker molecule for the attachment of drugs and proteins to AuNPs [21,22]; MAN is a carbohydrate that has been employed in the design of contrast agents for molecular imaging in cancer [5]; and cRGD has been widely employed for the active targeting of cancer cells [23]. The resulting information from this study will be of benefit in the development of biomodified nanomaterials and their potential applications.

## 2. Materials and Methods

### 2.1. Materials

AuNPs of 20 nm diameter in citrate buffer (SC@AuNPs), lipoic acid (ALA), hydrochloric acid (HCl), sodium hydroxide (NaOH), and N-(3-dimethylaminopropyl)-N′-ethylcarbodiimide hydrochloride (EDC) were obtained from Sigma-Aldrich (Mexico City, Mexico). The cRGD peptide (Cyclo(Arg-Ala-Asp-D-Phe-Lys-cCys) was acquired from Peptides international (Louisville, KY, USA). 2-aminoethyl 2,3,4,6-tetra-O-acetyl-α-d-mannopyranoside hydrochloride (MAN) was obtained from Synthose Inc., (Concord, ON, Canada). Deionized water (18.2 MΩ cm) was used in all experiments.

### 2.2. ALA@AuNPs

Three milliliters (1 nM) of SC@AuNPs was washed three times at 11,500 rpm (30 min) and resuspended in deionized water (pH 11). Then, 2.5 mL (1 nM) of washed nanoparticles was incubated with 250 μL of ALA (10 mM) at constant stirring (3000 rpm, 48 h at room temperature). The excess of ALA was removed by centrifugation (11,500 rpm, 30 min) and then resuspended in deionized water at pH 7 and adjusted to 1 nM of AuNPs.

### 2.3. MAN@AuNPs

For the preparation of the MAN@AuNPs conjugates, 1 mL (1 nM) of ALA@AuNPs, 2 μL of MAN (100 mg/mL, in ethanol), and 10 μL of EDC (40 mM, pH 6.5) were mixed and incubated at constant stirring (3000 rpm, 48 h). Then, the conjugate was washed three times in deionized water at pH 11 and left in basic hydrolysis for 24 h.

### 2.4. cRGD@AuNPs

For the preparation of the cRGD@AuNPs conjugate, 1 mL of SC@AuNPs (1 nM, pH 7) was mixed with 2 μL of cRGD (100 mg/mL) and incubated at constant stirring (3000 rpm, 24 h). Then, cRGD@AuNPs were washed three times in deionized water and left in water at pH 7.

### 2.5. Characterization

UV-Vis spectra (300–700 nm) were recorded in a Beckman Coulter DU-530 (Life science) with quartz cuvettes (1 cm). The hydrodynamic diameter and Z–potential were recorded in a Zetasizer Nano ZSP analyzer (HeNe laser (633 nm, 5 mW), Malvern, Worcestershire, UK). Fourier transform infrared spectroscopy coupled to ATR analysis were recorded on a Perkin-Elmer Spectrum 100 spectrometer (Perkin Elmer, Akron, OH, USA). For this analysis, samples were deposited by drop casting on the ATR crystal. The microscopy analysis was performed in a JEM-2010F FASTEM JEOL coupled to a NORAN energy dispersive spectrophotometer (EDS) operating at 200 kV. For this analysis, the conjugates (SC-AuNPs, ALA@AuNPs, MAN@AuNPs, or cRGD@AuNPs) were prepared by depositing a drop of the samples onto carbon-coated copper grids (Ted Pella, Redding, CA, USA) and allowed to dry at room temperature overnight. The recorded images were analyzed using the software ImageJ version 1.54d (NIH, Wayne Rasband), and the statistical analysis was performed with the Origin Pro 2023b software (Northampton, MA, USA).

STM measurements were carried out in a NaioSTM system (Nanosurf AG, Liestal, Switzerland). The analysis was carried out at room temperature using mechanically cut Pt/Ir tips of 0.25 mm diameter (Nanosurf AG, Liestal, Switzerland). For the analyses, 50 µL of each sample was drop-casted onto a freshly cleaved HOPG substrate (5 × 5 × 2 mm, grade ZYB). The deposited samples were incubated for 30 min, washed with 2 mL of deionized water, and dried overnight in a vacuum. The STM images were analyzed by using the Nanotec Electronica WSxM 5.0 (Madrid, Spain) software [24].

## 3. Results

**Figure 1:**
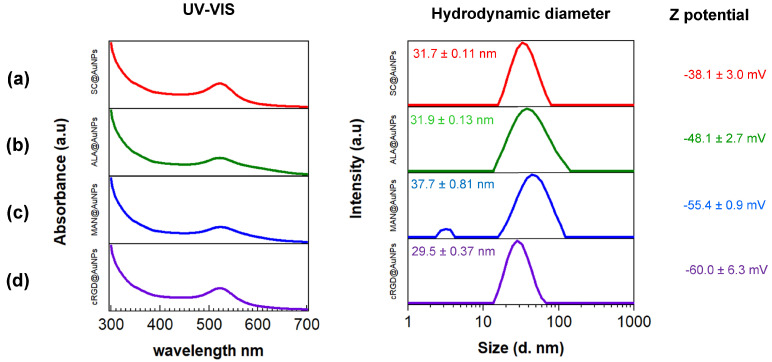
UV–Vis and DSL analysis of: (**a**) SC@AuNPs, (**b**) ALA@AuNPs, (**c**) MAN@AuNPs, and (**d**) cRGD@AuNPs. UV-VIS (left column), hydrodynamic diameter (middle column), and Z-potential (right column). All the measurements were conducted in deionized water at pH 11.0 by using NaOH (1 M). The color code of the surface coating is as follows: SC (red), ALA (green), MAN (blue), and cRGD (purple).

The UV–Vis spectroscopy results showed that SC@AuNPs and biofunctionalized samples ALA@AuNPs, MAN@AuNPs, and cRGD@AuNPs exhibited a strong absorbance band in the visible region at 523 nm without broadening, suggesting that the conjugates were stable after functionalization. The DLS measurement showed small differences; the diameters of SC@AuNPs and ALA@AuNPs were similar, 31.7 ± 0.11 nm and 31.9 ± 0.13 nm, respectively. After biofunctionalization with MAN, the size increased to 37.7 ± 0.81 nm, suggesting it was due to the covalent attachment of MAN. The size of the cRGD@AuNPs decreased to 29.5 ± 0.37 nm, a small change compared to the starting gold nanoparticles with sodium citrate. The size analysis showed that the conjugates and SC@AuNPs were moderately polydispersed (PDI 0.3–0.4); in particular, ALA@AuNPs and MAN@AuNPs showed a wider size distribution in comparison with SC@AuNPs and cRGD@AuNPs. Z potential analysis showed that the conjugates increased their negativity after biofunctionalization as follows: cRGD@AuNPs > MAN@AuNPs > ALA@AuNPs > SC@AuNPs (Figure 1, right column). These results suggest that the biomolecules contribute to the increased negativity. ALA can be attached to gold nanoparticles via the dithiol ring, with the carboxylic acid exposed to solution; the covalent binding of MAN to ALA increased the number of electronegative atoms on the AuNPs surface, and the binding of cRGD to AuNPs also increased the number of electronegative atoms on the surface.

**Figure 2:**
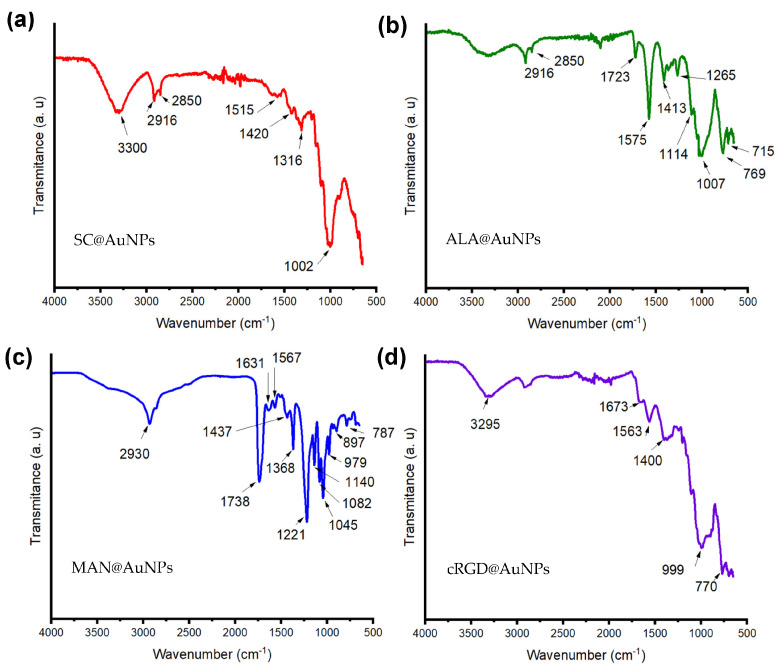
Attenuated total reflection Fourier transform infrared (ATR-FTIR) spectra of (**a**) SC@AuNPs, (**b**) ALA@AuNPs, (**c**) MAN@AuNPs, and (**d**) cRGD@AuNPs. The color code of the surface coating is as follows: SC (red), ALA (green), MAN (blue), and cRGD (purple).

To confirm the presence of biomolecules on AuNPs, FT-IR experiments were performed. The SC@AuNPs (Figure 2a) showed a peak at 3300 cm^−1^ that was assigned to OH stretching, one peak at 2916 cm^−1^ that was assigned to CH_2_, one peak at 1420 cm^−1^ assigned to carboxylic groups, and a peak at 1002 cm^−1^ assigned to CO; all these groups are present in sodium citrate [5]. The ALA@AuNPs (Figure 2b) showed one peak at 1730 cm^−1^ assigned to C=O double bond stretching, a peak at 2850 cm^−1^ assigned to CH_2_ symmetric stretching, and a peak at 2916 cm^−1^ assigned to CH_2_ asymmetric stretching; these peaks suggested the presence of ALA molecules [5]. After the covalent bond of MAN to ALA molecules attached to AuNPs, MAN@AuNPs (Figure 2c) showed a spectrum with three peaks at 1140, 1045, and 1082 cm^−1^ that suggested the presence of pyranose rings; meanwhile, the peak at 1738 cm^−1^ was assigned to the asymmetric vibration of C=O [25]. The peak at 1631 cm^−1^ was assigned to C=O and C-N stretching. Meanwhile, the peak at 1567 cm^−1^ suggested in-plane N-H bending and C-N stretching, and the C–O–C asymmetric frequency was placed between 1368 and 1045 cm^−1^. Finally, the peak at 1221 cm^−1^ was assigned to C-N stretching, and the peak at 1045 cm^−1^ was related to C-O stretching. The cRGD@AuNPs showed a peak at 3295 that was assigned to the N-H stretching of Amide A, one peak at 1673 cm^−1^ was assigned to the stretching band of C=O of Amide I, and the peak at 1563 cm^−1^ was assigned to the C-N stretching and N-H bending of amide II; meanwhile, the peak at 777 cm^−1^ was assigned to the out-of-plane N-H bending. As a whole, the FTIR analysis suggested the presence of each one of the biomolecules employed for biofunctionalization [26].

**Figure 3:**
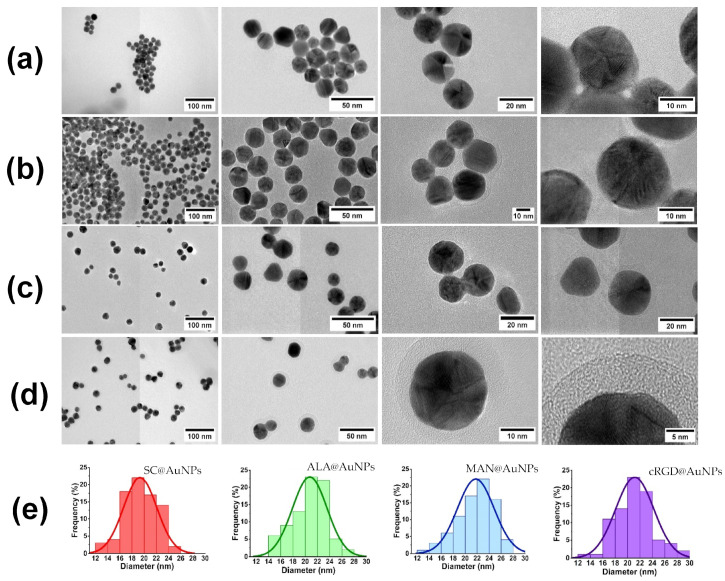
Sequences of transmission electron microscopy images and size histogram distribution for (**a**) SC@AuNPs, (**b**) ALA@AuNPs, (**c**) MAN@AuNPs, and (**d**) cRGD@AuNPs. (**e**) Histograms are indicated by the color code of the surface coating as follows: SC (red), ALA (green), MAN (blue), and cRGD (purple).

Transmission electron microscopy images of the biofunctionalized nanoparticles are shown in Figure 3. All the conjugates were spherical, and no changes in size and morphology occurred after biofunctionalization. The histograms of size distribution are shown in Figure 3e. However, there was the presence of a low-electron-density halo around ALA@AuNPs, MAN@AuNPs, and RGDc@AuNPs (see Figure 3b–d). This material was associated with the presence of ALA, MAN, and cRGD on the surface of the AuNPs. The size of the coat for ALA was near 1.07 nm, for MAN near 3.72 nm, and for cRGD 4.8 nm. The elemental analysis of the conjugates confirmed the presence of gold as well as N and S, and the gold was related to AuNPs; meanwhile, the other elements were related to ALA, MAN, and cRGD.

Although TEM imaging suggested the adsorption of ALA, MAN, and cRGD molecules onto the surfaces of the AuNPs, it did not allow us to resolve finer structural details. For this purpose, the STM technique was explored to analyze the conjugates. We used HOPG as a substrate for the analysis since it may have advantages over metallic substrates. It was reported that the thiolated protecting coat of gold nanoparticles can redistribute on the Au(111) surface in minutes and form islands [27]. Also, HOPG consists of high-quality defect-free graphene layers that provide clean and atomically flat surfaces suitable for material depositions, it is easily cleaved [17], and the defects are easy to recognize and differentiate from gold nanoparticles.

**Figure 4:**
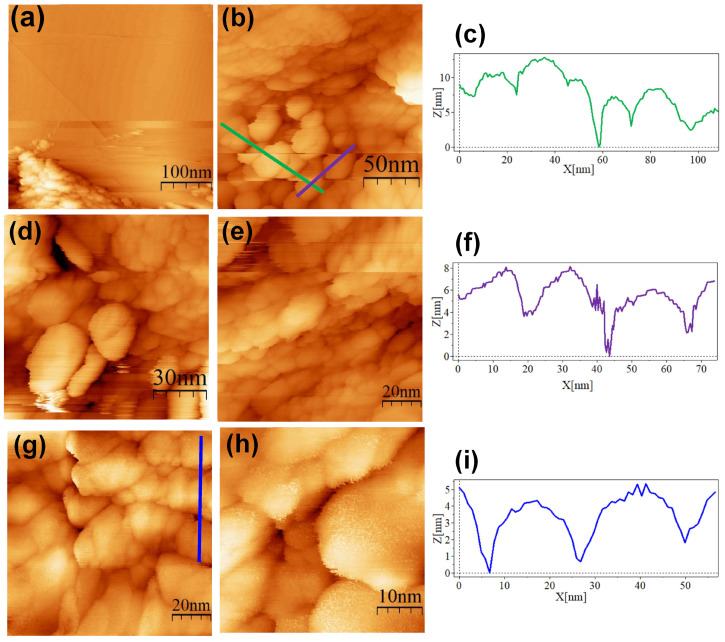
STM topographic images of SC@AuNPs deposited onto bare HOPG. STM imaging parameters: (**a**) (400 × 400 nm^2^, V*_s_* = 0.5 V, *I* = 1.0 nA, time/line: 0.382 s); (**b**) (181 × 181 nm^2^, V*_s_* = 0.5 V, *I* = 1.0 nA, time/line: 0.3 s); (**c**) profile corresponding to the blue line in (**b**); (**d**) (114 × 114 nm^2^, V*_s_* = 0.5 V, *I* = 1.0 nA, time/line: 0.3 s); (**e**) (103 × 103 nm^2^, V*_s_* = 0.5 V, *I* = 1.0 nA, time/line: 0.3 s); (**f**) profile corresponding to the green line in (**b**); (**g**) (103 × 103 nm^2^, V*_s_* = 0.5 V, *I* = 1.0 nA, time/line: 0.382 s); (**h**) (44.3 × 44.3 nm^2^, V*_s_* = 0.5 V, *I* = 1.0 nA, time/line: 0.3 s); (**i**) profile corresponding to the blue line in (**g**).

**Figure 5:**
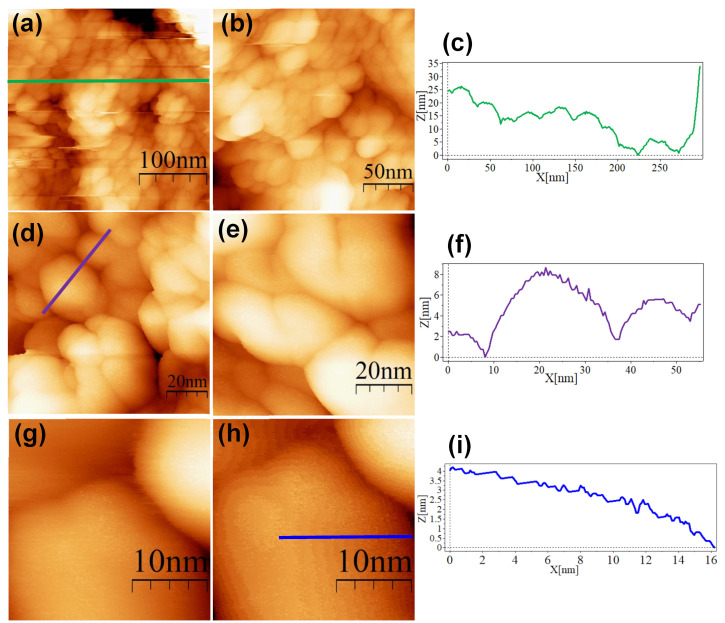
STM topographic images of ALA@AuNPs deposited onto bare HOPG. STM imaging parameters: (**a**) (300 × 300 nm^2^, V*_s_* = 0.5 V, *I* = 1.0 nA, time/line: 0.3 s); (**b**) (200 × 200 nm^2^, V*_s_* = 0.5 V, *I* = 1.0 nA, time/line: 0.3 s); (**c**) profile corresponding to the blue line in (**a**); (**d**) (97.3 × 97.3 nm^2^, V*_s_* = 0.5 V, *I* = 1.0 nA, time/line: 0.3 s); (**e**) (70.9 × 70.9 nm^2^, V*_s_* = 0.5 V, *I* = 1.0 nA, time/line: 0.3 s); (**f**) profile corresponding to the blue line in (**d**); (**g**) (26.6 × 26.6 nm^2^, V*_s_* = 0.5 V, *I* = 1.0 nA, time/line: 0.3 s); (**h**) (26.6 × 26.6 nm^2^, V*_s_* = 0.5 V, *I* = 1.0 nA, time/line: 0.3 s); (**i**) profile corresponding to the blue line in (**h**).

**Figure 6:**
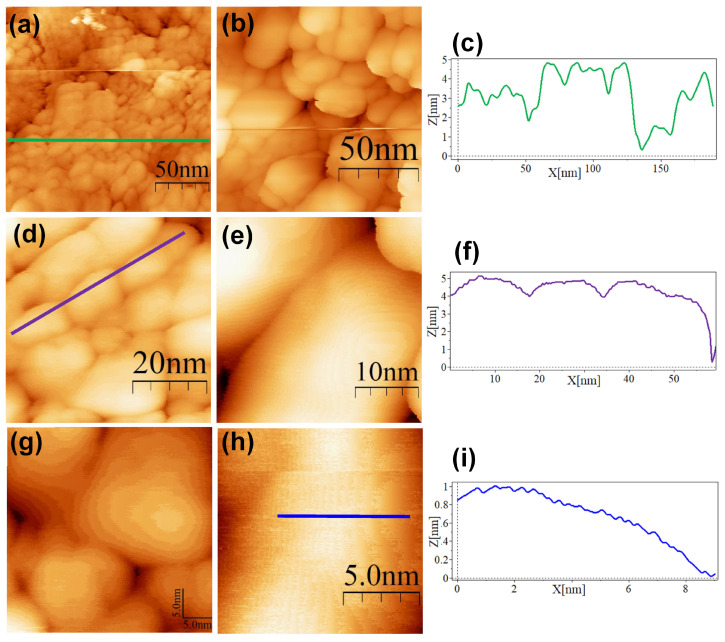
STM topographic images of MAN@AuNPs deposited onto bare HOPG. STM imaging parameters: (**a**) (189 × 189 nm^2^, V*_s_* = 0.5 V, *I* = 1.0 nA, time/line: 0.365 s); (**b**) (128 × 128 nm^2^, V*_s_* = 0.5 V, *I* = 1.0 nA, time/line: 0.365 s); (**c**) profile corresponding to the blue line in (**a**); (**d**) (54.7 × 54.7 nm^2^, V*_s_* = 0.5 V, *I* = 1.0 nA, time/line: 0.365 s); (**e**) (32 × 32 nm^2^, V*_s_* = 0.5 V, *I* = 1.0 nA, time/line: 0.365 s); (**f**) profile corresponding to the blue line in (**d**); (**g**) (35 × 35 nm^2^, V*_s_* = 0.5 V, *I* = 1.0 nA, time/line: 0.365 s); (**h**) (13.3 × 13.3 nm^2^, V*_s_* = 0.4 V, *I* = 1.0 nA, time/line: 0.365 s); (**i**) profile corresponding to the blue line in (**h**).

**Figure 7:**
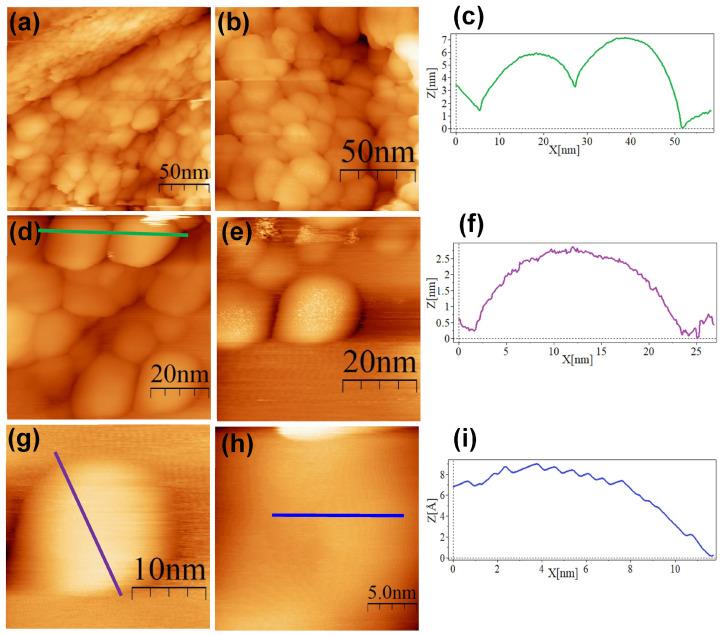
STM topographic images of cRGD@AuNPs deposited onto bare HOPG. STM imaging parameters: (**a**) (200 × 200 nm^2^, V*_s_* = 0.5 V, *I* = 1.0 nA, time/line: 0.4 s); (**b**) (134 × 134 nm^2^, V*_s_* = 0.5 V, *I* = 1.0 nA, time/line: 0.4 s); (**c**) profile corresponding to the blue line in (**d**); (**d**) (69.1 × 69.1 nm^2^, V*_s_* = 0.5 V, *I* = 1.0 nA, time/line: 0.399 s); (**e**) (54.6 × 54.6 nm^2^, V*_s_* = 0.5 V, *I* = 1.0 nA, time/line: 0.399 s); (**f**) profile corresponding to the blue line in (**g**); (**g**) (27.3 × 27.3 nm^2^, V*_s_* = 0.5 V, *I* = 1.0 nA, time/line: 0.399 s); (**h**) (20.4 × 20.4 nm^2^, V*_s_* = 0.5 V, *I* = 1.0 nA, time/line: 0.4 s); (**i**) profile corresponding to the blue line in (**h**).

The SC@AuNPs showed large agglomerates of densely packed particles, in addition to sparse individual nanoparticles. Before STM analysis, these particles were washed several times, and throughout the process, the stabilizing citrate was washed off, decreasing the repulsive forces which maintain the stability of gold nanoparticles. Cross-sectional analysis of these samples showed diameters between 18 and 22 nm (Figure 4c,f,i) and 3 and 5 nm high. A smooth surface was observed after analyzing the conjugates at high resolution. The images of the ALA@AuNPs showed densely packed nanoparticles with evident borders between the nanoparticles as compared to SC@AuNPs. These particles are 25 to 28 nm long and 3 to 6 nm high (see Figure 5c,f). The analysis of individual nanoparticles also showed the presence of terraces. In addition, the cross-sectional analysis of particles under these conditions showed that the surface was not smooth (see Figure 5i). It is important to mention that ALA@AuNPs exhibited an ellipsoid shape that is characteristic of gold nanoparticles on flat surfaces, as seen with STM, which was not as evident for the SC@AuNPs. The images of MAN@AuNPs showed a pattern similar to ALA@AuNPs; however, it was more evident, even at high magnifications (Figure 6d). These particles showed a size between 20 and 25 nm long and 1 and 2 nm high. Finally, the cRGD@AuNPs showed the presence of a large number of individual nanoparticles with dimensions of 10 to 22 nm long and 3 to 8 nm high (Figure 7). The images showed the ellipsoid form despite the densely packed aggregation of the particles in contrast to the SC@AuNPs and MAN@AuNPs.

## 4. Discussion

In this work, gold nanoparticles 20 nm in diameter were covered with thiolated biomolecules via S-Au bonds. This is a post synthesis methodology, well known as ligand exchange, where sodium citrate from the initial nanoparticles is exchanged by the thiolated molecules. It is important to mention that the exposed molecules at the interface determine the chemical and biological properties of the conjugates. Our results showed that the nanoparticles were stable after ALA, MAN, or cRGD modification, as confirmed by UV-Vis. The incorporation of functional groups provided by these molecules, as suggested by the FTIR analysis, can help to maintain the stability of these colloids in water, for example, allowing the formation of hydrogen bonds and electrostatic repulsion interactions. For biomedical applications, the stability of AuNPs is compulsory as it plays an important role in determining their toxicity [28]. Here, the stability was analyzed after synthesis and in water at pH 11. The size of the AuNPs after functionalization showed small changes due to the adsorption of the biomolecules compared to the initial AuNPs stabilized with sodium citrate (SC@AuNPs). In contrast, the Z potential showed that the conjugates increased their negativity after biofunctionalization: cRGD@AuNPs > MAN@AuNPs > ALA@AuNPs > SC@AuNPs. The change in the electronegativity of the conjugates can be attributed to the presence of aspartic acid amino acids (D) for cRGD, electronegative oxygens for MAN, and carboxylic acids for ALA. The size and charge of the nanoparticles are significant parameters as it was reported that these physicochemical parameters can influence the in vitro and in vivo behavior of functionalized gold nanoparticles, i.e., the in vitro uptake and the in vivo biodistribution [29].

Our TEM images showed the presence of a low-density coating around ALA@AuNPs, MAN@AuNPs, and cRGD@AuNPs, suggesting the presence of the biomolecules [30]. In addition, no aggregates were observed, which indicates that the nanoparticles’ modification state diminished their aggregation behavior. On the other hand, our STM analysis showed that it is a viable microscopy technique for the characterization of biofunctionalized AuNPs. The STM images showed an aspherical morphology that was more evident for ALA@AuNPs and cRGD@AuNPs than for SC@AuNPs and MAN@AuNPs. The presence of free material could affect the morphology resolution in the MAN@AuNP case, the bonding reaction required mannose and EDC in excess, and even after several centrifugation washes, some residual material could be present, affecting the imaging of individual nanoparticles. Also, in the SC@AuNPs case, the presence of free molecules of sodium citrate could affect the resolution of individual molecules. The size of the nanoparticles measured by cross-sectional analyses showed particles with diameters longer than 20 nm, which can be attributed to the biomolecules attached to the AuNPs, as seen by TEM. A high-resolution analysis of the biomolecular coating of our as-synthetized conjugates was not achieved in our study. STM is a very sensitive technique, and several factors can limit its resolution power when analyzing biofunctionalized gold nanoparticles: the presence of free material can contaminate the tip, degrading its ability to produce images of high resolution; the mobility of the nanoparticles is another parameter that can affect an STM study. In our analysis, the mobility of individual nanoparticles on the HOPG substrate made it difficult to analyze their surfaces at a high resolution. Previous studies have reported the attachment of AuNPs on gold surfaces, which improved their analysis [15]. In addition, we employed the drop-cast technique for deposition, and we observed the formation of large agglomerates; in contrast, the deposition of monolayers can help to resolve individual AuNPs at a high resolution. In general, the previously mentioned parameters should be considered for the analysis of gold nanoparticles functionalized with biomolecules. Finally, the analysis of individual biomolecules as lipids, carbohydrates, proteins, or nucleic acids onto flat substrates is a complex task, and, moreover, the analysis of biomolecules on the surface of AuNPs represents an extreme challenge [17].

## 5. Conclusions

The biofunctionalization of AuNPs with lipoic acid, mannose, and the cRGD peptide via the formation of the Au-S bond via ligand exchange is a reliable method to obtain conjugates with potential biomedical applications. Combining several analytical techniques, the as-synthetized conjugates are described as stable without evidence of aggregation. Our preliminary studies using STM demonstrate that this technique can be employed to analyze biofunctionalized AuNPs; however, several parameters, such as the elimination of free ligands, the reduction in mobility, and the deposition of monolayers, should be optimized for the analysis of the surfaces of bioconjugates at a high resolution. The biofunctionalized nanoparticles with lipoic acid, mannose, and the cRGD peptide can also be conjugated with drugs and/or contrast agents. Also, it was shown that the STM can be used as a complementary technique for the characterization of biofunctionalized nanoparticles. Furthermore, the STM is a versatile technique that can be used for the electronic characterization of conjugates and the analyst of samples in physiological solutions.

## Data Availability

Not applicable.
